# Progressive Encephalomyelitis With Rigidity and Myoclonus With Thymoma: A Case Report and Literature Review

**DOI:** 10.3389/fneur.2020.01017

**Published:** 2020-09-18

**Authors:** Yana Su, Li Cui, Mingqin Zhu, Yixuan Liang, Ying Zhang

**Affiliations:** Department of Neurology and Neuroscience Center, The First Hospital of Jilin University, Changchun, China

**Keywords:** progressive encephalomyelitis with rigidity and myoclonus, thymoma, anti-glycine receptor antibody, anti-glutamic acid decarboxylase antibodies, tumors

## Abstract

Progressive encephalomyelitis with rigidity and myoclonus (PERM) is part of the variant type of the Stiff Person Syndrome (SPS) and is a rare neurological disease. We report here a patient with PERM who had thymoma and was positive for anti-glutamic acid decarboxylase (anti-GAD) antibodies. Her symptoms improved after treatment with hormones and gamma globulin. We also summarized the literature review of patients with PERM accompanied by tumors reported.

## Introduction

Stiff person syndrome is a rare neurological disease, mainly manifested by axial and limb muscle stiffness, and muscle painful spasms especially after tactile noise, and emotional stress stimulation. Moersch and Wohman (1) first reported in 1956 under the name “Still-man Syndrome.” Progressive encephalomyelitis with rigidity and myoclonus is a variant of stiff person syndrome. Its main clinical symptoms are in addition to the typical SPS symptoms, as well as sensory, brainstem symptoms (ataxia, vertigo), spinal cord symptoms, and autonomic symptoms, most patients have anti-glutamic acid decarboxylase (anti-GAD) antibodies, some patients found anti-glycine (GlyR) antibodies. In addition, PERM can be accompanied by tumors, such as thymoma (2–5), Hodgkin's lymphoma (6–8), small cell lung cancer (9, 10), breast cancer (11), etc. Here we report a case of GAD antibody- positive PERM associated with thymoma.

## Case Presentation

A 60-year-old female patient was admitted to the hospital on May 21, 2019 due to “unstable walking and stiff legs for more than 10 days.” The patient had instability during walking more than 10 days before admission. Stiff bilateral lower limbs appeared at the time of walking, showing muscle tension in the lower limbs, affecting walking, and falling in severe cases, which gradually eased after about 1 min, and fell 5 times during the course of the disease. Accompanied by itching of the right lower extremity, mainly hip. The above symptoms aggravate after tension, emotional excitement, and touch. The patient denied trauma, infection, poisoning, drugs, psychiatric disease, and family genetic history.

On admission, she was alert and well-oriented. Admission examination of the nervous system: small horizontal nystagmus in both eyes; increased muscle tension in both lower extremities; marked rigidity of her lower limbs were also noticedactive tendon reflexes; hyperreflexia was observed in the extremities, and ankle cramps in both legs. There were no sensory disturbances.

Laboratory tests: blood routine, urine routine, liver and kidney function, blood lipids, ions, surgical comprehensive, and tumor markers were normal. Thyroid function: T3, T4 normal, TSH: 6.67 uIU/ml, antithyroid peroxidase antibody: 96.51 uIU/mL (<35), antithyroglobulin antibody: 272.5 uIU/mL (<115); IG antibody 18 U/L (0–12); anti-SSA-60 +, granular type 1: 100, anti-SSA-52/Ro52 +, anti-mitochondrial M2 antibody +, high-sensitivity C-reactive protein 6.03 (0–3.5 mg/L). Cerebrospinal fluid analysis showed no abnormality. The serum anti-GAD antibody IgG was positive and anti-GAD antibody in cerebrospinal fluid was not detected. Anti-islet cell antibody also was positive. Antibodies to Amhiphysin, Yo, Hu, Ri, CV2, Ma2, PCA-2, NMDA receptors, and VGKC were negative in serum. Myasthenia gravis antibodies also were negative. Unfortunately, We were not able to measure anti-glycine antibody titers in our patient due to limited conditions. Neostigmine test: negative.

**Figure 1:**
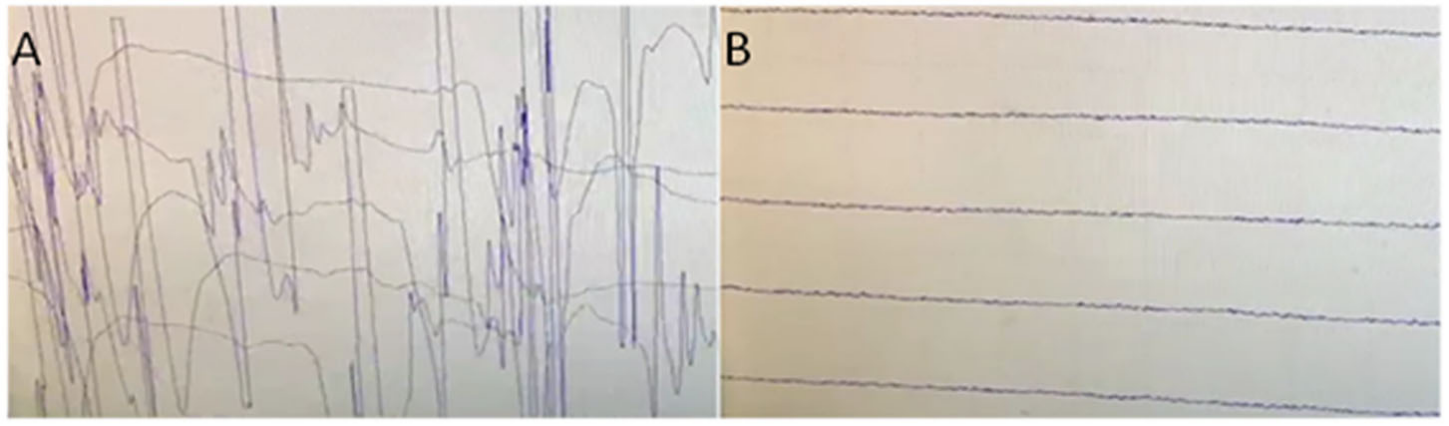
**(A)** Electromyographic examination: continuous motor unit activity (CMUA)was seen. **(B)** After the injection of 10 mg of diazepam, the CMUA gradually weakened and disappeared.

Head MRI only showed right lacunar infarction. Spinal cord MRI was normal. Electromyographic examination: continuous motor unit activity (CMUA) was seen (Figure 1A). After the injection of 10 mg of diazepam, the CMUA gradually weakened and disappeared (Figure 1B). CT of the thorax revealed an anterior superior mediastinal mass, suggestive of a thymoma.

After admission, pregabalin (75 mg, 2/day) and baclofen (10 mg, 3/day) were given orally, but the symptoms did not improve. And the patient started difficulty water, dysphagia 10 days after admission. So much that a feeding tube was inserted 15 days after admission, binocular dyskinesia suddenly appeared, and eyes paralyzed to the right. Muscle stiffness and myoclonus in both lower extremities gradually increased, and itching in the right lower extremity was unbearable. To some extent, the patient also presented emotional irritability and anxiety. On examination, she was anxious with no evidence of cognitive deterioration. According to the medical history and auxiliary examination results, she was diagnosed with PERM supported by the clinical diagnosis criteria for SPS proposed by Dalakas et al. (12).

After taking the diagnosis of PERM into consideration, the patient was treated with intravenous methylprednisolone therapy (80 mg/day for 15 days) followed by maintenance therapy with oral methylprednisolone (60 mg/day). She also received intravenous immune globulin (20g/day) for 3 days. Clonazepam taken orally before bedtime. The patient's eye muscle paralysis disappeared after a few days, there was obvious relief of stiffness of lower limbs, paroxysmal myoclonus, difficulty swallowing, and itching and pain symptoms. Discharged neurological examination (2019-06-26): small horizontal nystagmus in both eyes, gastric tube removal, mild stiffness, and cramps in the lower limbs. Hyperreflexia was still observed in the extremities, and ankle cramps in right leg. The patient's thymoma had surgery indications. In view of the obvious improvement of symptoms and economic reasons, the patient and her family decided to postpone the operation.

After discharge, the methylprednisolone was gradually reduced orally, and clonazepam 1 mg was orally maintained before going to bed. On 2020-1-7 telephone follow-up, the patient experienced another episode of dysphagia and had to keep the gastric tube in the past 20 days. Later, the symptoms gradually improved with the increase of clonazepam dose. Recently, on 2020-7-15 telephone follow-up the patient had difficulty in swallowing again, and her lower limbs were so stiff that she dared not walk independently. As far as the patient's current situation is concerned, we recommend again that the removal of the thymoma and immunotherapy if possible.

## Discussion

In 1976, Whiteley et al. (13) first reported two cases of encephalomyelitis associated with stiff-man syndrome (SPS) and subacute myoclonic spinal neuronitis, which had been classified as stiff-man superposition syndrome, and later confirmed to be progressive encephalomyelitis with rigidity and myoclonus (PERM). With people's understanding of the disease, PERM is found to be a severely life-threatening autoimmune disease characterized by rigidity, muscle pain and spasm, deep and shallow sensory disturbances and symptoms of the brainstem spinal cord, autonomic function, dyspnea, and stimulus-induced myoclonus. The patient here we reported had stiff muscles of the lower extremities, with paroxysmal myoclonus, itching, and pain; the course of the disease gradually develops, difficulty swallowing so severe that need nasal feeding, and paralysis of both eyes to the right; emotional changes and touch stimulation can induce or exacerbate the above symptom. Electromyographic examination: continuous motor unit activity can be seen, found in rectus abdominis, thoracolumbar paravertebral muscle, and proximal lower limb. After the injection of 10 mg diazepam, the CMUA gradually weakened and disappeared. These clinical symptoms and positive results for anti-GAD antibodies were consistent with PERM. Though there is still no clear diagnostic criteria for PERM. The patient was also accompanied by thymoma. The patient's symptoms improved after treatment with hormones, immunoglobulins, clonazepam, etc. Unfortunately, the patient did not perform thymectomy due to economic reasons and improved symptoms, and the patient experienced relapse of dysphagia, which may be related to the persistent cause of thymoma not removed.

Several neurologic disorders with similar clinical presentations should be considered, including neuromyotonia (NMT), tetanus, and psychogenic dyskinesia. Neuromyotonia is a clinical syndrome characterized by muscle peristalsis, fasciculation, muscle spasm, rigidity, and hyperhidrosis caused by a variety of reasons. Its muscle rigidity is characterized by no relief during sleep, The limbs are more common and rarely involve rectus abdominis. Typical EMG: continuous motor unit activity, slowed movement and sensory conduction, and no response to diazepam, all of which are obviously different from PERM. Tetanus usually has a history of trauma, with masseter muscle spasm as the first symptom. The typical symptom is opisthotonus, and strong muscle spasm caused by external stimuli, ranging from a few seconds to a few minutes each time. It can be distinguished from PERM by the history of trauma, typical symptoms, and the ineffectiveness of tranquilizers, stiff muscles during sleep, and the EMG CMUA can't be completely suppressed by tranquilizers. Psychogenic dyskinesia is mainly manifested as attacks began and ended abrutly, complex and variable manifestations, there are distraction of attention and entrainment effects and co-activation of the affected limb. There are many psychological factors, suggesting or placebo treatment may be effective. Anti-GAD antibodies and EMG are helpful for identification.

The cause of PERM is not clear and may be related to immunity. In recent years, it has been reported that it may be related to anti-glycine receptor (GlyR) antibody. Glycine receptor is a pentameric ligand-gated chloride channel. In the adult nervous system, they are mainly found in the spinal cord and brain stem, which mediate rapid inhibition (14). Antibodies targeted at Glycine receptor may lead to a persistent startle response. Gamma-aminobutyric acid (GABA) is an inhibitory neurotransmitter. Glutamate decarboxylase(GAD) can catalyze the conversion of the excitatory neurotransmitter glutamic acid to GABA. Antibodies against GAD may affect functioning of GABA-ergic neurons (15). All of antibodies target proteins of GABAergic and glycinergic inhibitory synapses, except for dipeptidyl-peptidase-like protein-6 (DPPX)-antibodies (16). DPPX antibodies are a regulatory subunit of the Kv4.2 potassium channels on the surface of neurons, which are involved in somatodendritic signal integration and attenuation of back-propagation of action potentials. Patients with DPPX antibodies tend to have hyperekplexia, prominent cerebellar ataxia with marked eye movement disorder, trunk stiffness, sensory disturbance, and gastrointestinal symptoms. Our patient had anti-GAD antibodies which can explain the excessive neuronal activities resulting in the typical clinical symptoms. However, Our case simultaneously had many other antibodies but not meet the disease diagnosis criteria, such as antithyroid peroxidase antibody, anti-islet cell antibody, antithyroglobulin antibody, anticardiolipin IgG antibody, anti-SSA-60, granular type, anti-SSA-52/Ro52, anti-mitochondrial M2 antibody in her serum, which indicated the involvement of immune dysfunction. SPS and PERM are associated with other autoimmune conditions including diabetes mellitus, thyroiditis, autoimmune thyroid disorders, and pernicious anemia. Kraemer et al. reported a case of PERM that hypothyroidism was diagnosed 3 months before first motor symptoms and that both, the Hashimoto's thyroiditis and the diabetes mellitus were of recent onset (17). Therefore, it is still necessary for our patient to surveillance the development of potentially autoimmune diseases, such as thyroiditis and diabetes. The mechanisms of interaction between these antibodies need further investigation.

We summarized the cases of PERM reported in recent years (Tables 1, 2) and found that there are more men than women, and the reason for this difference is unknown. The age of onset varies, the age range at presentation is 14 months−81 years with a mean of 50 years. Patients with tumors are generally older. Patients have a variety of onset symptoms. Approximately 35% of patients have onset of muscle stiffness and spasms, and 25% of patients have onset of brainstem symptoms. However, the clinical manifestations are mainly stiff spasms of the trunk and limb muscles, accompanied by brainstem spinal cord symptoms, including eye movement paralysis, gaze paralysis, nystagmus and drooping eyelids, difficulty swallowing, and difficulty articulating. In general, patients will have obvious autonomic symptoms such as sweating, tachycardia, and urinary retention and even autonomic failure, which may include respiratory failure, and must be managed in intensive care. Twenty-five percentage of the patients require mechanical ventilation and mortality up to 40% (42). In most patients, there is no abnormality of the head and spinal cord magnetic resonance. MRI of one patient showed increased signal intensity throughout the length of the cervical cord and lower brainstem on the T2 weighted scan and reduced signal intensity in this region on the Tl weighted scan (20). MRI with diffusion-weighted and fluid-attenuated inversion recovery (FLAIR) weighted sequences (WS) of one patient showed left temporal and insular cortical hyperintensities without gadolinium enhancement (39). MRI of the head in 2 patients showed cerebellar atrophy (23, 33). MRI can differentially diagnose other diseases. EMG has diagnostic significance, and the emergence of CMUA is one of the important diagnostic criteria.

**Table 1:** Eleven reported cases of PERM associated with tumors; demographics, clinical features, investigation results, management, and outcome.

**References**	**Uehara et al. (2)**	**Clerinx et al. (3)**	**Morise et al. (4)**	**Ozaki et al. (5)**	**Current case**	**Schmidt et al. (7)**	**Borellini et al. (6)**	**Tchapyjnikov et al. (8)**	**Spitz et al. (9)**	**Kyskan et al. (10)**	**De Blauwe et al. (11)**	**Shugaiv et al. (18)**
**DEMOGRAPHIC**												
Age at onset	52	49	72	75	60	21	60	18	73	39	66	46
Sex	Female	Male	Female	Female	Female	Male	Male	Male	Male	Male	female	Male
**CLINICAL FEATURES**												
Initial symptoms	Awkwardness in her left leg	Pain in his right leg	Dysarthria and chewing difficulties with masticatory fatigue	Progressive difficulty in walking and rigidity of the lower extremities	Stable walking, stiff legs	Generalized pruritus, paroxysmal fear, and disturbance of sleep	Low back pain and progressive rigidity of the trunk and lower limbs	Progressive difficulty walking and several falls	Muscular spasms on the left lower limb	A locked left jaw and leg myoclonus	Inability to look to the left and gait instability	Diplopia, dysphagia, and gait ataxia
Myoclonus	+	+	+	+	+	+	+	+	+	+	+	+
Stiffness	+	+	+	+	+	+	+	+	+	–	–	+
Spasm	+	+	+	+	–	+	+	+	+	+	+	+
Brain stem sign	–	Speech and swallowing difficulties intermittent diplopia	Abnormalities in ocular movements, dysarthria, and dysphagia	Dysarthria, dysphagia	Difficulty water, dysphagia	Bilateral ptosis and mydriasis, hypometric saccades	Dysphonia Dysphagia	Dysphagia	Dysphagia	Difficulty swallowing food and could only open his mouth Dysarthria	A bilateral horizontal gaze palsy, hypoesthesia	Diplopia, dysphagia
Nueropsychi atric symptom	+	–	–	–	–	+	–	+	_	_	_	_
Dysautonomia	–	Dry mouth, constipation urinary retention, and excessive sweating	-	Urinary retention, constipation, hyperhidrosis, hypersalivation, and sinus tachycardia	–	Tachyarrhythmia, hyperthermia, hyperhidrosis, hypersalivation, and paralytic ileus	Hyperhydrosis, constipation, and urinary retention	Urinary retention	–	–	–	–
MRI	Normal	Normal	Normal	Normal	Normal	Normal	Normal	Normal	Normal		Normal	Normal
**ANTIBODIES**												
GAD (serum/CSF)	+/+	−/−	+/+	+/+	+/na	−/na	−/−	−/na	+/na	−/na	−/na	−/na
GlyR (serum/CSF)	na/na	+/na	+/+	+/+	na/na	+/na	+/+	na/na	na/na	+/na	+/na	na/na
AchR	–	na	+	–	–	–	na	-	na	-	na	na
Amphiphysin	–	–	–	–	–	–	na	-	na	–	–	na
Other Abs	–	–	Anti-thyroid peroxidase and anti-thyroglobulin antibodies	TPO Abs	–	–	-	Antimicrosomal and antithyroglobul-in antibodies	–	–	-	–
Tumor type	Thymoma	Thymoma	Thymoma	Thymoma	Thymoma	Hodgkin's lymphoma	Hodgkin's lymphoma	Hodgkin's lymphoma	Lung cancer	Lung cancer	Breast cancer	Renal cell carcinoma
Management	PLEX thymectomy	Methylprednisolone, diazepam, and baclofen thymectomy	IVIG PLEX Corticosteroids Thymectomy	PLEX thymectomy	IVIG Corticosteroids Diazepam Baclofen Clonazepam	Corticosteroids Lorazepam Tiagabine ABVD	Corticosteroids PLEX Clonazepam Gabapentin ABVD	IVIG CorticosteroidsPLEX Lorazepam	IVIG Diazepam Phenytoin	Corticosteroids PLEX IVIG Lorazepam Baclofen Rituximab	Corticosteroids PLEX IVIG Carbamazepine Diazepam	Corticosteroids azathioprine left nephrectomy
Relapse	No	No	Yes	No	Yes	No	No			Yes		
Outcome	She fully returned to her ordinary daily living, which has now persisted for 1 7 years, without relapse or deterioration	At the time of manuscript submission, the patient remains free of neurologic symptoms and is farming at his predisease level	Her left horizontal gaze palsy and psychiatric symptoms improved, and her MMSE improved to 22/30	Died after suffering a cerebral infarction	On 2020–1-7 telephone follow-up, the patient experienced another episode of dysphagia in the past 20 days	The patient was able to return to school and had no limitations in his daily life	Over a 12-month follow-up period, a constant clinical improvement was observed despite tapering of immunosuppressive and symptomatic treatment	The patient's mental status normalized and he showed dramatic improvements in his leg and truncal spasticity as well as dysphagia		His symptoms completely resolved after treatment	She was too weak for any additional treatment and eventually passed away August 2012	His mRS remained at 3 in a follow-up period of 2 years under azathioprine treatment

*GAD, glutamic acid decarboxylase; GlyR, glycine receptor; AchR, acetylcholine receptor; Abs, antibodies; CSF, cerebrospinal fluid; IVIG, intravenous immunoglobulin; PLEX, plasma exchange; TPO, thyroid peroxidase; na, not available*.

**Table 2:** Other reported cases of PERM without tumors; demographics, clinical features, investigation results, management and outcome.

**Authors**	**Age/Sex**	**Clinical features**	**MRI**	**Antibodies**	**Management**	**Outcomes**
Whiteley et al. (13)	61/F	Stiffness of her legs and difficulty in walking, shaking of her legs from time to time, and of precipitancy of micturition			Levodopa, diazepam, orphenadrine, baclofen dimethothiazine	She died 13 months after the onset of her illness
Whiteley et al. (13)	19/M	Aching pain in his back and legs, weakness of his legs and difficulty in walking, brief attacks of rigidity and jerking of his limbs, slurred speech and difficulty swallowing after 13 days				Twenty-one days after the onset of his illness, he was found dead on the floor beside his bed
Howell et al. (19)	56/F	Vertigo and vomiting, severe burning pain in the left side of the neck and head, painful spasms, gait instability, difficulty in chewing, and swallowing			Dexamethasone diazepam	Died after a complex illness lasting 26 months
McCombe et al. (20)	52/F	Three months of progressive neurological disturbance. Pain in the interscapular region, shoulders and chest, numbness of the hands and arms, weakness of the upper and lower limbs and urinary retention, episodes of muscle spasms	Abnormal		Carbamazepine baclofen, sodium valproate, and phenytoin	During a trial of withdrawal of anticonvulsant therapy, after 3 months of treatment, the muscle spasms returned
Burn et al. (21)	50/F	Weakness in her upper limbs, stiffness, and loss of range of movement in her neck, double vision, mild dysphagia and a strangled quality to her voice, painful muscle spasm	MRI of the head and spine was normal	GAD+ AchR+ gastric parietal auto-antibody+ thyroid thyroglobulin and anti-TMAb +	Diazepam immunosuppressive therapy has not been used	Since her initial admission in September 1989 the patient's disabling spasms and rigidity have slowly worsened
Kullmann et al. (22)	39/M	Dysphagia, dysarthria, nausea, pain in the throat and neck, and positional vertigo, facial, and nuchal jerks after 2 weeks, myoclonus	MRI of the head with gadolinium enhancement were normal	Positive gastric parietal and islet cell antibodies	Carbamazepine sodium valproate clonazepam midazolam	Seven years after presentation, he continues to have mild generalized myoclonic jerks
Kullmann et al. (22)	34/M	Disturbance of taste, anorexia, and insomnia, 4 weeks later he developed dysphagia, painful jerks, muscular rigidity and myoclonus, tachycardia, hypertension, and mydriasis	Repeated brain and spinal cord MRI was again normal		Corticosteroids PLEX Clonazepam Baclofen Sodium valproate	Subsequent to discharge, all his abnormal signs resolved, stimulus-sensitive jerks recurred on attempting to discontinue clonazepam
Back et al. (23)	27/M	Cerebellar ataxia and dementia, followed by rigidity and oculomotor dysfunction	Abnormal		Corticosteroids, Azathioprine, tizanidin, carbamazepine baclofen, diazepam	He died 33 months after onset of symptoms
Kraemer et al. (17)	81/F	Severe rigidity, stiffness and superimposed muscle spasms, tachycardia, hyperhidrosis and arterial hypertension, urine and stool incontinent, moderate dysphagia	The MRI of the brain and the whole spine showed no abnormalities	GAD+ TPO+	IVIG Corticosteroids Benzodiazepines baclofen, vigabatrin	A few months later her gait had suddenly become more unsteady. The rigidity of the right leg had worsened
Saidha et al. (24)	60/F	Paraspinal muscle rigidity, muscle spasms and new onset scoliosis, ataxic gait with dysmetria and deteriorating mobility. Scanning dysarthria, dysmetria, and intention tremor	MRI of brain and spine were normal	GAD+,ICA+,TPO+ AAA+, anti-transglutaminase, and anti-endomysial antibodies +	Corticosteroids PLEX,IVIG Methotrexate, azathioprine, Mycophenolate mofetil, Infliximab, rituximab Benzodiazepines	Dysarthria also improved, as did mobility and she was able to walk using cross bars
Hutchinson (25)	54/M	Left flank tingling and 2 weeks of worsening brief frequent violent jerks, spontaneous, and triggered by sensory and auditory stimuli; Four weeks after admission he developed mild bilateral ptosis, bilateral partial horizontal gaze palsies	Cranial and spinal MRI scans with gadolinium were normal	GlyR+	Corticosteroids PLEX Cyclophosphamide Levetiracetam Clonazepam	Now he is stable with mild spinal rigidity, walks 200 m with one stick, and works part time; horizontal gaze is normal
Mas et al. (26)	33/F	Diplopia, dysphagia, and gait ataxia, progressively developed rigidity of lower limbs with painful spasms, involuntary jerks and contracture of both ankles and urinary retention	Brain and spinal MRI and was normal	GlyR+	Corticosteroids IVIG Diazepam and baclofen	She has been asymptomatic for 8 years
Mas et al. (26)	60/M	Dysphagia, diplopia, left facial palsy and right trigeminal hypoaesthesia, muscular rigidity, corticospinal signs, myoclonic jerks, and severe dysautonomia	Brain MRI were normal	GlyR+		He has remained in a persistent vegetative state and ventilator-dependent
Mas et al. (26)	48/M	Leg rigidity and frequent spells of trismus, muscle spasms followed by opisthotonus and diaphoresis. Progressively more aggressive with emotional irritability. Dysgeusia (metallic taste) and severe concurrent behavioral changes and diurnal hypersomnia	Brain and spinal MRI scans were normal	GlyR+	Corticosteroids IVIG Diazepam gabapentin	His leg stiffness was partially improved but persistence of the pruritus, dysgeusia, hypersomnia, masseter spasms with yawn, and behavioral changes
Turner et al. (27)	28/M	Seizures and erectile dysfunction 3 weeks later urinary retention, jerky eye movements, ataxia, limb rigidity with myoclonus	MRI of the brain and whole spinewere normal.	GlyR+ NMDAR+	Levetiracetam	Death within days of admission from multi-organ failure
Iizuka et al. (28)	61/F	Reduced sense of taste, A week later she began to feel stiffness, back pain, then myoclonic jerks and painful spasms dysphagia,dysarthria, left gaze palsy, right-gaze evoked counterclockwise rotatory nystagmus, and slow saccade to the left	Brain, cervical, thoracic, and lumbar MRIs were normal	TPO+ Tg-Abs	IVMP, IVIg, Corticosteroids Cyclosporine Baclofen, diazepam Clonazepam	Initially improved but twice relapsed (40 months)
Peeters et al. (29)	37/M	A 1-month-history of muscle jerks, painful spasms, falls, diplopia, dysphagia, incomplete jaw opening, constipation, urinary retention, and pruritus of the arms and back	Brain and spinal MRI were normal	GlyR+	Corticosteroids PLEX Diazepam baclofen	Upon discharge neurological exam was normal, apart from mild hypertonia of the limbs and slightly slowed upward saccades
Shugaiv et al. (18)	54/M	Difficulty walking and profuse sweating, generalized stiffness, rigidity and upward gaze restriction, he had right central facial palsy, spastic paraparesis, myoclonic jerks, and startle response	Cranial and spinal MRI scans were normal	None	Corticosteroids IVIG	The patient did not respond to 7 pulse steroid and intravenous IVIg treatment courses performed on several occasions
Shugaiv et al. (18)	67/M	Amnesia, left focal motor seizures. Within 2 months, generalized rigidity, stiffness, myoclonic jerks, dysphagia, and mutism followed by stupor	Cranial and spinal MRI scans were normal	GAD+ LGI1+ VGKC+	Corticosteroids IVIG Anticonvulsants	He has not developed new symptoms in a follow-up period of 3 years
Joana et al. (30)	14months./M	Irritability, restless sleep, and sudden episodes of axial hyperextension, rigidity, and generalized myoclonus. Soon after, she developed laterocollis to the right, left hemifacial spasm, trismus, and urinary retention		GlyR+	Corticosteroids IVIG Levetiracetam Clonazepam	The paroxysms disappeared 10 days after starting immunotherapy
Stern et al. (31)	40/M	Prodrome of mood disturbance and myoclonic jerks. One week later presented with rigidity, ophthalmoplegia, myoclonus, sweating	Magnetic resonance (MR) brain imaging were normal	GlyR+	IVIG PLEX Corticosteroids clonazepam Baclofen	Gradual response but relapsed at 7 months
Wuerfel et al. (32)	3/M	Generalized epilepsy, Seizures, tamper tantrums, headaches, and sleep disturbance	Cranial MRI at 3.0 Tesla was normal.	GlyR+	Corticosteroids lamotrigine	
Balint et al.(33)	16/M	Unsteadiness of the right arm and excessive startle, scoliosis, thoracic spine, trunk stiffness	MRI of the brain was normal	DPPX+	IVIG, PLEX Corticosteroids Rituximab Benzodiazepines	At follow-up 21 months after symptom onset, normal walking was unimpeded, but tandem gait and standing on one leg were impossible
Balint et al. (33)	27/M	Muscular stiffness, jerky spasms after 4 years constipation developed, blurred vision on external gaze and acrophobia. recently, paraesthesia and allodynia	MRI of brain and spine were normal	DPXX+	IVIG Corticosteroids Rituximab Clonazepam	The clinical course was stable for 2 years with the abovementioned treatment regimen
Balint et al. (33)	26/M	Unclassified ocular motor disturbance and hyperhidrosis. By then, broken pursuit and gaze-evoked nystagmus, trunk stiffness, Jerks, slurred, and scanning speech as well as gait and limb ataxia. Intense allodynia and neurogenic pruritus, memory and attention deficits, hyperreflexia, urinary retention	MRI of the brain and the spine were initially normal. Seventeen years after onset, MRI of the brain disclosed mild cerebellar atrophy	DPPX+	IVIG, PLEX Corticosteroids Cyclophosphamide	Due to dysphagia, he recurrently contracted severe pneumonia requiring intensive care treatment, which eventually led to death
Bourke et al.(34)	55/M	24 months of progressive stiffness and pain in his legs, jerks of his entire body and painful extensor spasms of his limbs and trunk. then hyperekplexia	MRI of the brain was normal.	GlyR+	IVIG,AzathioprineCorticosteroidsClonazepam, diazepamphenytoin, baclofen	Five years after presentation he was living independently with moderate rigidity, a slow and stiff gait
Bourke et al. (34)	58/F	stiffness in the legs, body jolts, loss of consciousness	Cranial and spinal MRI with were normal	GlyR+	Corticosteroids Methotrexate Diazepam Clonazepam	She has remained in remission
Seguier et al. (35)	73/F	Abdominal pain,then hypophonia and dyspnea, cognitive impairment and stuttering, muscle cramps	normal		IVIG Diazepam Corticosteroids Benzodiazepines Rituximab	
Ueno et al. (36)	48/F	Spasticity of the lower limbs and subsequently developed upper limb spasticity, perioral myoclonus and restlessness after 3 months, dysautonomia	MRI of the brain/pelvis were normal	None	IVIG Levetiracetam azathioprine Diazepam	Patient showed a dramatic improvement following immune moderation
Kenda et al. (37)	67/M	Four weeks history of speech and swallowing difficulties, leg weakness, and shortness of breath. Twitching of his face and limb muscles	Brain MRI was unremarkable	GlyR+	Immunoadsorption PLEX Levetiracetam azathioprine Diazepam	One year from symptom onset, he suffered from disease relapse
Degeneffe et al. (38)	62/F	Lower back pain, lumbar muscular contractures, and back rigidity. Then lower limb, myoclonus, confusion, hyperthermia, and acute respiratory failure	Brain and spinal magnetic resonance imaging (MRI) revealed a pituitary adenoma	GAD+	IVIG PLEX Corticosteroids Levetiracetam	Seven months after admission, she had no relapse of the myoclonus nor the rigidity and was able to walk without assistance
Wirth et al. (39)	61/M	within a few days, right eyelid ptosis and diplopia. Two weeks later, he experienced widespread painful spasms, multifocal stimulus-sensitive myoclonus followed by hypertonic tetraparesis, swallowing difficulties, somnolence, and respiratory failure	Abnormal	GlyR+ VGKC+ LGI1+ CASPR2+	IVIG Corticosteroids Botulinum toxin Cyclophosphamide Diazepam Baclofen	When reevaluated 3 months later, the patient remained stiff in all four limbs, but was nonetheless able to walk
Witek et al. (40)	55/F	Intellectual disability, immobility, and inability to swallow safely after 1 week, mild bifacial weakness, profound axial and limb rigidity, severe dysarthria, and polyminimyoclonus in her jaw and distal aspects of her extremities	Normal	GAD65+	IVIG Corticosteroids Benzodiazepines Rituximab	Months after her initial presentation, she has not had any signs of a relapse of her symptoms
Jazebi et al. (41)	65/M	Two week history of hand tremor, abnormal movements, slowed mentation, weakness, and poor balance. Within 3 days of admission, cerebellar ataxia, infrequent non-rhythmic multifocal myoclonic appendicular and facial jerks, coarse intention tremor, unsteady standing and posture, and increased muscle tone with rigidity	Normal	GAD+	IVIG PLEX	

*Boxes left blank when data unavailable*.

*AchR, acetylcholine receptor; GAD, glutamic acid decarboxylase; DPPX, dipeptidyl-peptidase-like protein 6; NMDAR, N-methyl-D-aspartate receptor; GlyR, glycine receptor; VGKC, voltage-gated potassium channel; LGI1, leucine-rich glioma inactivated 1; CASPR2, contactin-associated protein 2; IVIG, intravenous immunoglobulin; TMAb, thyroid microsomal autoantibodies; ICA, Anti- islet cell antibodies; AAA, anti-adrenal cortex antibody; PLEX, plasma exchange; TPO, thyroid peroxidase*.

The incidence rate of tumor in our statistics are about 20%, which is consistent with the previous literature report (43). We summarized these 12 cases in Table 1. There were 5 cases with thymoma (2–5), 3 cases with Hodgkin's lymphoma (6–8), 2 cases with lung cancer (9, 10), 1 case with breast cancer (11), 1 case with kidney cancer (18). All 7 patients who tested for GlyR antibodies were positive for GlyR antibodies. Almost all patients tested for GAD antibodies, but less than half of the patients were positive for GAD antibodies. Among the 12 patients with tumors, patients with glycine antibody positive were more likely to have dysautonomia.

A retrospective analysis recently found GlyR antibodies in patients of PERM, some of these also had GAD antibodies, DPPX antibodies (33), NMDAR antibodies (27). Patients with GlyR antibodies often have prominent brainstem involvement, and often sensory and autonomic symptoms. GAD antibodies associate with palatal myoclonus, epilepsy, sporadic progressive ataxia. Patients with DPPX antibodies tend to have hyperekplexia, prominent cerebellar ataxia with marked eye movement disorder, trunk stiffness, sensory disturbance, and gastrointestinal symptoms. Multiple antibodies found in some patients of PERM. Therefore, it is difficult to predict the specificity of these antibodies in PERM based on clinical evidence. Further research is needed to clarify each of their roles in PERM. When we consider that a patient may be diagnosed with PERM, we should screen for related antibodies as much as possible.

The treatment of PERM mainly includes symptomatic treatment and immunotherapy. There were 5 patients with thymoma among the patients with tumor, 4 of whom responded well to thymectomy. There were 3 patients with Hodgkin's lymphoma among the patients with tumor, 2 of whom responded well to ABVD chemotherapy. Patients with lung cancer, breast cancer, and kidney cancer have a poor prognosis. The stabilization and recovery may occur if the underlying tumor is detected and treated early. Therefore, in the early stage of diagnosis, tumors should be screened comprehensively, irrespective of the serology in all patients. Most cases of PERM without tumors showed substantial and sustained improvement with immunotherapies, usually with combinations of corticosteroids, IVIg, and plasma exchange, and Rituximab has been reported to have a significant effect.

We reported a patient with PERM who had thymoma and was positive for anti-GAD antibodies, and also summarized the current literature on clinical characteristics, investigation results, management, and outcome of patients of PERM. At the same time, this case also has some limitations, such that we can't measure the anti-glycine antibody titers due to limited lab conditions. In addition, the patient did not perform thymectomy due to economic reasons that maybe result in the patient experienced relapse of dysphagia after being discharged. When encountering similar clinical manifestations, it will be better to test related antibodies as comprehensively as possible and actively advise patients to perform surgical treatment to remove the original lesion to prevent recurrence. We hope that this report will provide a basis for further understanding of PERM and mention clinical doctors better identify and treat these similar presentations so as not to delay diagnosis and treatment.

PERM is a complex autoimmune disease that requires a clinician to diagnose it in combination with the patient's symptoms and signs and auxiliary examinations. With the in-depth study of PERM and the further development of detection technologies such as GAD antibodies and anti-glycine antibodies, people have gradually deepened their understanding of PERM disease, but the diagnosis still lacks specific antibodies. Further investigation is needed to uncover the nature of disease.

## Data Availability Statement

All datasets generated for this study are included in the article/supplementary material.

## Ethics Statement

Written informed consent was obtained from the individual(s) for the publication of any potentially identifiable images or data included in this article.

## Author Contributions

YS and LC participated in writing the manuscript. MZ and YL participated in collecting the information of the paper and analysis or interpretation of data. YZ participated in revising the manuscript. All authors contributed to the article and approved the submitted version.

## Conflict of Interest

The authors declare that the research was conducted in the absence of any commercial or financial relationships that could be construed as a potential conflict of interest.
